# The “tweaking principle” for task switching

**DOI:** 10.1186/1471-2202-15-S1-P14

**Published:** 2014-07-21

**Authors:** Salva Ardid, Xiao-Jing Wang

**Affiliations:** 1 Department of Neurobiology and Kavli Institute for Neuroscience, Yale University, New Haven, Connecticut 06510, USA; 2 Department of Biology and Centre for Vision Research, York University, Toronto, Ontario, Canada, M3J 1P3; 3 Center for Neural Science, New York University, New York, New York 10003, USA

## 

A hallmark of executive control is the brain’s agility to shift between different tasks depending on the behavioral rule currently in play [1]. Humans and other animals exhibit a remarkable ability to flexibly select an appropriate response to a sensory input, and rapidly switch to another sensory-response mapping when task rule or goal changes. An increasing number of monkey experiments have been performed using task-switching paradigms, combined with single-neuron recording from sensory, parietal, and prefrontal cortical areas. Physiological evidence from these studies suggests that modulation of neural activity by task rule is typically weak [2]. By contrast, most previous models commonly assume that a rule signal is similarly strong as sensory stimulation in affecting activity of cortical neurons [3]. How can small rule modulation explain large (binary) behavioral changes in task switching?

In this work, we propose a solution to this puzzle, which we refer to as “the tweaking hypothesis” [4]. The core idea is that network reconfiguration underlying task switching can be realized by very weak top-down signals from rule neurons in prefrontal cortex. This is because a weak input can be greatly amplified through reverberating “attractor” dynamics in categorization and decision circuits, ultimately leading to circuit selection in favor of one sensory-motor mapping over another.

We tested the tweaking hypothesis by developing a neural circuit model for task switching that consists of several basic and interacting circuit modules for sensory coding, rule representation, categorization of stimulus features, and action selection, respectively [4]. The model was validated by reproducing salient single-neuron physiological observations [2] and behavioral effects associated with task switching [1,5,6]. Notably, the model identifies specific circuit mechanisms, in terms of neural dynamics and reward-dependent synaptic plasticity, that explain salient and widely observed behavioral effects associated with task switching [4]: (i) Switch cost: response time and error rate increase in trials following a task switch. Switch cost splits into a component that decreases with a longer time for preparation and a residual component that remains [5]. (ii) Task-response interaction: on task repeat trials, the response time is shorter if the same motor response is repeated; by contrast on switch trials, response time is shorter if an alternative motor response is selected [5,6]. (iii) Congruency effect: response times and the error rate are larger when the stimulus is incongruent compared to when it is congruent, which depends on whether the mapped behavioral response is different or the same, according to alternative rules [5,6].

This work represents a neural circuit model for task switching and sheds insights in the brain mechanism of a fundamental cognitive capability; in particular, that category-selective neurons play an essential role in resolving the sensory-motor conflicts that typically appear in task-switching paradigms [4].
